# Heat treatment effect on the mechanical properties, roughness and bone ingrowth capacity of 3D printing porous titanium alloy

**DOI:** 10.1039/c7ra13313h

**Published:** 2018-04-03

**Authors:** Zuhao Li, Chang Liu, Bingfeng Wang, Chenyu Wang, Zhonghan Wang, Fan Yang, Chaohua Gao, He Liu, Yanguo Qin, Jincheng Wang

**Affiliations:** Department of Orthopedics, The Second Hospital of Jilin University Changchun 130041 P. R. China heliu@ciac.ac.cn jinchengwang@hotmail.com qinyanguo@hotmail.com; School of Materials Science and Engineering, Central South University Changsha 410083 P. R. China; Department of Orthopedics, Hallym University 1 Hallymdaehak-gil Chuncheon Gangwon-do 200-702 Korea

## Abstract

The weak mechanical strength and biological inertia of Ti–6Al–4V porous titanium alloy limit its clinical application in the field of orthopedics. The present study investigated the influence of different solution temperatures (*e.g.* 800 °C, 950 °C and 1000 °C) on the mechanical properties, roughness and bone ingrowth capacity of Ti–6Al–4V porous titanium alloy prepared by Electron Beam Melting. It was found that the compressive and shear strength were promoted with the increase of solution temperature because of the transformed crystallinity of Ti–6Al–4V titanium alloy and phase changes from TiAl to TiAl + TiV. In addition, the topological morphology, surface roughness and wettability of the porous titanium alloy scaffolds were improved after heat treatment, and in turn, the adhesion rate and cell proliferation of bone marrow mesenchymal stem cells were enhanced. Compared with the scaffolds before and after heat treatment at 800 °C, the scaffolds heat-treated at 950 °C and 1000 °C achieved better bone ingrowth, extracellular matrix deposition and osseointegration. These findings indicate the great potential of heat treatment in possessing Ti–6Al–4V porous titanium alloy for orthopedic implant.

## Abstract 2

The weak mechanical strength and biological inertia of Ti–6Al–4V porous titanium alloy limit its clinical application in the field of orthopedics.

## Graphical abstract

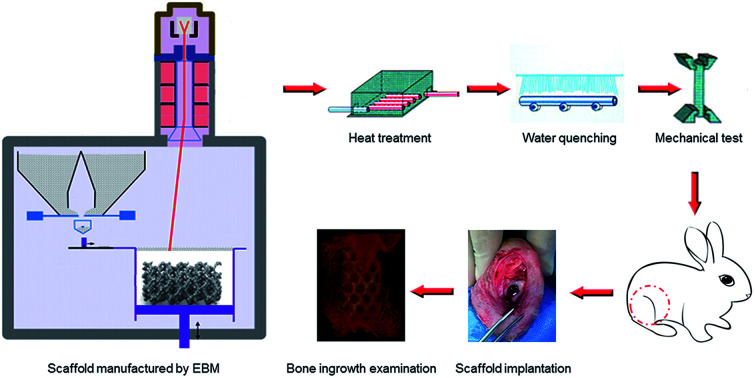

## 1. Introduction

Severe fractures, tumors and infections causing large bone defects are tricky problems in the field of orthopedics. Once the bone defect exceeds the critical size, it cannot be repaired through self-regeneration.^1^ The traditional clinical resolutions for treatment of bone defect include autologous and allogeneic bone graft, and follow-up reported good clinical outcomes.^2,3^ Autologous bone graft is the gold standard clinical material for bone regeneration in term of osteoconduction and osteoinduction. However, there are many disadvantages remaining, such as limited bone source, high infection rate and secondary surgery. As for the allogeneic bone graft, the problems of low biocompatibility, expensive cost and disease transmission still perplex the surgeons^4,5^.

Three dimensional printing (3D printing) technology can fabricate the personalized anatomical implants using titanium alloy (mainly Ti–6Al–4V) to realize the perfect reconstruction of bone defect for patients depending on the digitally precise design, which have been demonstrated by our previous works.^6,7^ Meanwhile, 3D printing techniques can build complex structures inside the implant and adjust the geometry parameters to promote bone ingrowth, thereby significantly enhance implant–bone interface stability and prevent prosthesis loosening and sinking.^8,9^ More importantly, adequate interconnectivity and porosity of microporous structure can be designed and obtained. These properties can increase the contact area of implant and surrounding bone, and enhance the initial stability of the interface.^10,11^ However, although the porous features facilitate cellular migration and proliferation as well as nutrients and oxygen transportation required for vascularization, the mechanical strengths of the porous scaffolds have been demonstrated to decrease with the increasing porosity, which is especially important for fulfilling the bone defect in the bearing area.^12–14^

Various chemical and physical methods have been investigated to improve the mechanical strength (*e.g.* shear and compressive strengths) of porous titanium alloy.^15,16^ Heat treatments on titanium alloy have been investigated extensively, and attempts have been made to model the phase morphology, microstructure and mechanical properties.^17,18^ With the increase of solution temperature, grain boundary, phase boundary within the material phase heterogeneous interface can affect mechanical properties of weak links. On the other hand, in the case of heat treatment, the grain size and crystallization of the material will change, and also affect the mechanical properties of the material.^19,20^ Hence, suitable heat treatment on porous titanium alloy to achieve desired mechanical properties for medical application is worthy trying. However, studies on influence of heat treatment on biocompatibility and bone ingrowth of porous titanium alloy are rarely reported.

**Scheme 1:**
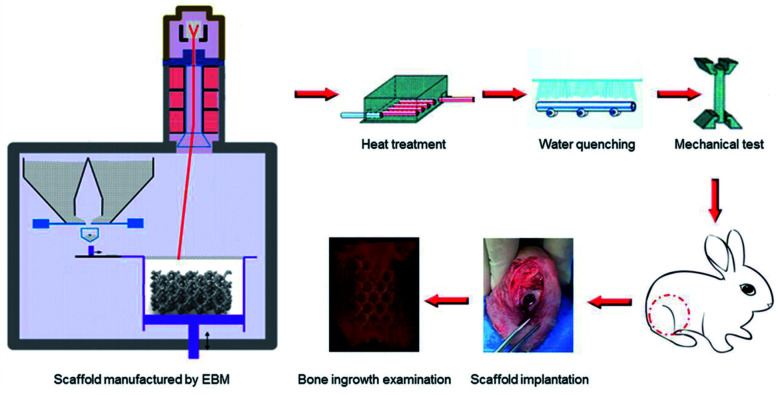
Schematic illustrations of the heat treatment procedures and the potential influence on mechanical properties as well as bone ingrowth in a rabbit bone defect model.

In this paper, porous titanium alloy scaffolds were prepared by Electron Beam Melting (EBM) as the pre-designed type. Then, scaffolds experienced solution treatment at 800 °C, 950 °C and 1000 °C, and then water quenching. The microstructure and mechanical properties between original and different heat-treated scaffolds were compared. In addition, the biocompatibility *in vitro*, such as cellular adhesion and proliferation, as well as *in vivo* osseointegration experiment of all samples in the rabbit bone defect models were also investigated Scheme 1.

## 2. Materials and methods

### 2.1. Materials

Ti–6Al–4V powder (grade 23) was provided by AK Medical Co., Ltd. (Beijing, P. R. China). Kroll's reagent was bought from Aladdin Reagent Co., Ltd. (Shanghai, P. R. China). Van Gieson's picrofuchsin and calcein AM staining solution were purchased from Sigma-Aldrich (Shanghai, P. R. China). Low glucose Dulbecco's modified Eagle's medium (LG-DMEM) and fetal bovine serum (FBS) were bought from Gibco (Grand Island, NY, USA). Cell Counting Kit-8 (CCK-8) for mammalian cells was obtained from Beyotime Co., Ltd. (Shanghai, P. R. China).

### 2.2. Preparation of 3D printing Ti–6Al–4V alloy scaffold

Spherical pre-alloyed Ti–6Al–4V powder was used for manufacturing porous titanium alloy scaffolds by EBM (Q10, Arcam, Sweden) as described detailedly in ref. 21. The porous scaffolds were based on dodecahedron unit cells with the following design (nominal) dimensions: strut size = 300 mm, pore size = 800 mm, porosity = 70%. Disk-shaped scaffolds (*∅*10 mm × *L*3 mm) were used for phase transformation, microstructure and cellular biocompatibility assays, and the columnar-shaped scaffolds (*∅*5 mm × *L*10 mm) were used for mechanical and *in vivo* bone ingrowth investigations. Furthermore, disk-shaped scaffolds (*∅*10 mm × *L*1 mm) without porosity were used for contact-angle analysis.

### 2.3. Processing conditions of heat treatment

Disk-shaped and columnar-shaped scaffolds experienced solution treatment in argon atmosphere at 800 °C, 950 °C and 1000 °C for 1 h according to previous transition thermodynamics data (the β transus temperature was 995 °C),^22^ and then water quenching. Finally, 30% HNO_3_ alcohol solution was used to remove oxide films from the alloy surface for 1 min.

### 2.4. Analysis of phase transformation

Phase analysis was performed using an X Ray Diffractometer (XRD) (D500, Siemens, Germany) to analyze the lattice constant and grain sizes, using CuKα radiation at 40 keV and 40 mA from 10 to 80 degrees of 2*θ*. In addition, the as-processed scaffolds were also characterized by differential scanning calorimetry (DSC) using a differential thermal analyzer (STA-499C, Netzsch, German), applying a heating rate of 10 K min^−1^, the test temperature was beginning from room temperature to 1200 °C at a speed of 10 °C S^−1^ temperature rise in an argon atmosphere.

### 2.5. Analysis of microstructure change

The scaffolds were performed the polishing with metallographic sandpaper on the fine grinding, and then, optical microstructure characterizations of the scaffolds were analyzed. In brief, specimens were mirror polished using different diamond suspensions and proper disks, and then polished with a mixture of colloidal silica and hydrogen per-oxide (30%). Specimens were then chemically attached by Kroll's reagent (1 mL HF, 3 mL HNO_3_ and 80 mL H_2_O) and quickly washed in ethanol to remove residual etchant. Finally, the scaffolds were flattened and the optical microstructures were observed and analyzed by optical microscope (POLYVAR-MET, Reichert-Jung, Austria). Besides, the fracture face microstructures and surface roughness of the scaffolds were also analyzed *via* scanning electron microscopy (SEM) (Nova400, FEI, USA) as previous described in ref. 23. In addition, the topological morphology and quantitative analysis of surface roughness were obtained by atomic force microscopy (AFM) (Nanoscope V multimode 8 SPM, Bruker) as described previously in ref. 24. In brief, the scaffolds were measured by AFM in intermittent contact mode matched with a silicon nitride cantilever (spring constant: 0.4 N m^−1^ and scan rate: 1 Hz). The topological morphologies were displayed on the computer with three dimensional morphologies and then photographed. Furthermore, average roughness values (*R*_a_) and root mean square values of surface roughness (*R*_q_) were selected to evaluate the roughness of the sample surface.

### 2.6. Analysis of contact-angle

The surface wettability of the sample was represented by the contact angle, which was detected *via* static sessile-drop technique by goniometer (Ramé-Hart). In brief, water droplet from a hypodermic needle was placed onto the scaffold surface, and the image was recorded after stabilization. The angle between sample surface and tangents of droplet were measured.

### 2.7. Mechanical performance testing

The compressive strength and shear strength, which are the most important mechanical properties for medical application, of columnar-shaped scaffolds (*n* = 3, each test) were tested using material mechanics testing machine (Instron3369, Instron Corporation, USA). Before test, the specimen was placed in a steel mold, and then axial and lateral forces were applied to the scaffold respectively. The compressive and shear strength as the displacement of the specimens under external force were recorded.

### 2.8. *In vitro* cell experiments

#### 2.8.1. Cellular adhesion and morphological analysis

Isolation and culture of bone marrow mesenchymal stem cells (BMMSCs) was performed as previous described in ref. 25, and the 3rd generation of BMMSCs was readied for use. To detect the adhesion effectiveness of BMMSCs on different heat-treated scaffolds, the scaffolds were sterilized under ultraviolet-irradiation light for 30 min, and then put into 24-well plates. Afterwards, 500 μL of BMMSCs suspensions in LG-DMEM medium supplemented with 10% (v/v) FBS were added onto the scaffolds with a density of 5 × 10^4^ per well. After 24 h of culture, the scaffolds were washed with cold phosphate buffer saline (PBS) for 3 times, a qualitative adhesion assay was performed using CCK-8 kiting. In brief, 300 μL of CCK-8 solution in LG-DMEM medium with 10% FBS (v/v) was added to each well at predetermined time intervals. After incubation for 4 h, the absorbance at 450 nm and 630 nm (baseline correction) were measured with a Bio-Rad microplate reader (Model 550, Hercules, CA, USA). At the same time, the morphological analysis of BMMSCs attached on the scaffolds was also detected by calcein AM cell staining. In brief, BMMSCs adhered on scaffolds were incubated in PBS (0.01 = M, pH = 7.4) containing 2 mM calcein AM at 37 °C for 30 min. Cells were then observed by using a fluorescent microscope and images were captured, and viable cells were stained green with calcein AM.

#### 2.8.2. Cell proliferation on scaffolds

Different heat-treated scaffolds were sterilized under ultraviolet-irradiation light for 30 min. Afterwards, the scaffolds were put into 24-well plates, and the BMMSCs suspensions (1.0 × 10^6^ cells) were added onto each scaffold. 1 mL of LG-DMEM medium supplemented with 10% (v/v) FBS was then added and replaced every three days. The proliferation of BMMSCs was assessed by CCK-8 method at predetermined periods (*i.e.*, 1, 7, 14 days).

### 2.9. Bone ingrowth and osseointegration performance *in vivo*

#### 2.9.1. Implantation of the scaffolds

Bone defect models of New Zealand white rabbits (2.7–3.0 kg, 5–6 months old, *n* = 24) were carried out for animal experiment. All animal procedures were performed in accordance with the Guidelines for Care and Use of Laboratory Animals of Jilin University and approved by the Animal Ethics Committee of Jilin University. In brief, rabbit was anesthetized with 3% (w/v) pentobarbital at a dosage of 50 mg kg^−1^, and then the patellar groove of left keen joint was exposed *via* medial parapatellar approach after the skin preparation and sterilization, and the lateral subluxation of patella was performed. The rabbits underwent drilling for bone defect (diameter = 5 mm and depth = 4 mm), and then the original and heat-treated scaffolds (*n* = 6 × 4 = 24) were transplanted into the defects. The patella was replaced, and the wound was closed in layers. Postoperatively, the rabbits were allowed free movement, and penicillin with a dosage of 1.50 mg kg^−1^ was injected intramuscularly to prevent infection each day in the following 3 days.

#### 2.9.2. Micro-CT examination

After 12 weeks after implantation, the animals were executed and all the specimens of the left femoral condyle were obtained. To observe the effect of bone growth into the scaffold, the specimens were subjected to micro-CT examination, and the quantitative analysis of bone volume and bone volume/total volume (BV/TV) were obtained. To estimate the bone ingrowth quality, trabecular thickness and trabecular spacing of the specimens were also acquired.

#### 2.9.3. Detaching test of the simples

A standard push-out test for the scaffolds (*n* = 3, each group) was used for shear strength evaluation of the bone-implant interface,^26^ and the remaining scaffolds (*n* = 3, each group) were used for histological examination. Briefly, careful cleanness of the periosteum before the test was performed to expose the implant. Then, the specimens were displaced on the plate for detaching test at 1.5 mm min^−1^ using a closed-loop servo-hydraulic testing machine (MTS MiniBionix, Minneapolis, USA). The load at which the scaffolds became detached from the bone was recorded as the maximum load.

#### 2.9.4. Histological examination

The implants and adjective bone were harvested and fixed in 10% formalin for 2 weeks and dehydrated in graded ethanol (40%, 75%, 95%, and 100%) for 2 days, and then embedded in methyl methacrylate. Thin slices (150–300 μm) were cut from the blocks and ground to a thickness of 40 to 50 μm using transverse saw cuts and a polishing machine (exact band saw; Exact Apparatebau, Norderstedt, Germany), and each section was then stained with Van Gieson's picrofuchsin, which the purple, green and silver indicate bone, fibrous tissue and titanium alloy scaffold, respectively. Histological evaluation was performed on the stained sections using a digital microscope (DSX 500; Olympus Corporation, Japan).

#### 2.9.5. Elemental analyses

Elemental analyses of calcium (Ca) and phosphorus (P) of regenerated bone tissue was performed as described in ref. 27. In brief, 30 mg of bone powder, which was previously powdered by cryogenic mill (CertiPrep 6750 Freezer/Mill, SPEX), was dissolved in a 10 mL solution of 10% HNO_3_ and 3% H_2_O_2_, and digested by microwave. The concentrations of Ca and P were measured by an Optima 8300 ICP-OES (Perkin Elmer).

### 2.10. Statistical analyses

The data were expressed as means ± standard deviation (SD) and the statistical analysis were carried out using ANOVA with Tukey's post-hoc analysis (SPSS Inc., Chicago, IL, USA). *P* < 0.05 was considered statistically significant difference.

## 3. Results and discussion

### 3.1. 3D printing porous titanium alloy scaffolds

**Fig. 1:**
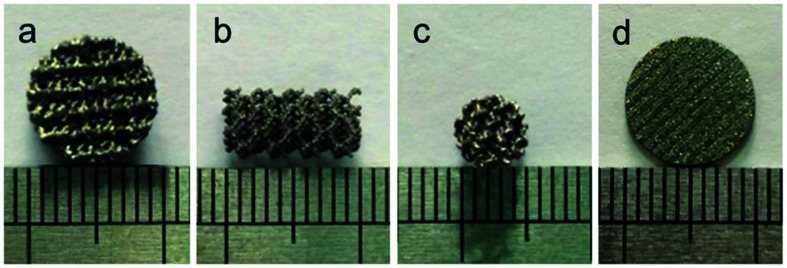
Disk-shaped scaffolds (*∅*10 mm × *L*3 mm (a), *∅*10 mm × *L*1 mm (d)) and columnar-shaped scaffolds (*∅*5 mm × *L*10 mm, (b) and (c)) prepared by EBM.

Disk-shaped scaffolds (*∅*10 mm × *L*3 mm Fig. 1a, *∅*10 mm × *L*1 mm Fig. 1d) and columnar-shaped scaffolds (*∅*5 mm × *L*10 mm, Fig. 1b and c) were successfully prepared by EBM, and the scaffold surface behaved titanium metal luster with uniform pore distribution.

**Fig. 2:**
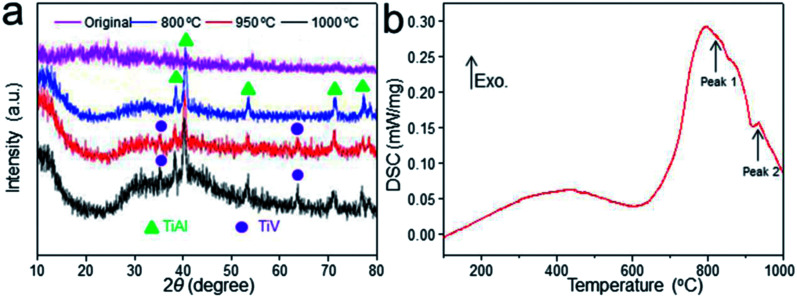
XRD patterns of the scaffolds before and after heat treatment (a). DSC profile of the thermal effect of heat treatment on Ti–6Al–4V titanium alloy scaffolds (b).

### 3.2. Characterizations of porous titanium alloy scaffold after heat treatment

The XRD profile of Ti–6Al–4V titanium alloy scaffolds was detected as shown in Fig. 2a. The characteristic “steamed bun” diffraction peak of the original scaffold was observed in 2*θ* = 25–35°. While, the diffraction peaks of TiAl phase of scaffold heat-treated at 800 °C could be observed in 2*θ* = 38.7°, 40.3° and 54.2°. In addition to strong TiAl crystallization peaks, some weak crystalline diffraction peaks could also be detected in 2*θ* = 35.4° and 63.9° with the temperature increase to 950 °C, indicating the intermetallic compound TiV. Meanwhile, stronger diffraction peaks of scaffold heat-treated at 1000 °C were observed. It could be concluded that Ti–6Al–4V prepared by EBM underwent phase changes from TiAl to TiAl + TiV with the increase of solution treatment temperature.

The DSC analysis was applied to explore the thermal effect of heat treatment on Ti–6Al–4V titanium alloy scaffolds as shown in Fig. 2b. The curves showed that the scaffold had an endothermic peak at 739.4 °C with heat absorption for 185.2 J g^−1^ (from 620.8 °C to 850 °C). This may be due to the occurrence of crystallization resulting to heat release. Moreover, the scaffold had another obvious endothermic peak at 940.2 °C with a heat absorption for 0.8 J g^−1^ (from 880 °C to 970 °C), which coincided with the phase transition temperature of the titanium alloy as shown in Fig. 2.

**Fig. 3:**
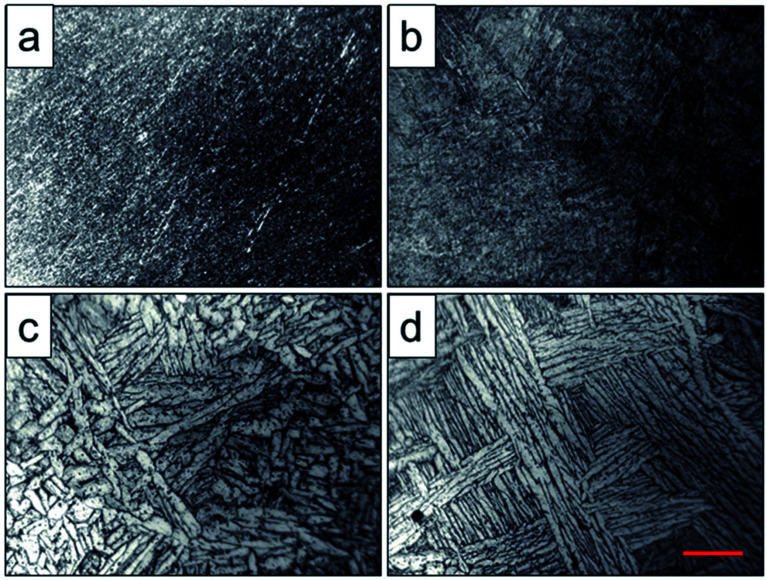
Optical microanalysis of original scaffold (a) and scaffolds heat-treated at 800 °C (b), 950 °C (c), 1000 °C (d) obtained by optical microscope, and the red scale bar = 50 μm.

Optical micrographs of the scaffolds after heat treatment were shown in Fig. 3. It showed that the phase particles were too fine to be observed of the original scaffold and scaffold heat-treated at 800 °C (Fig. 3a and b), which was lower than the phase transition point. As depicted in Fig. 3c, the grains became coarser, larger and flaky due to the temperature increase, and the primary α phase was transformed into β phase, while the β phase precipitated from the lamellar α phase. When the solution temperature reached 1000 °C, α phase particles were observed with lamellar arrangement and distributed in the metastable β phase particles. The α lamellar microstructure forming a net organization in β phase grains witch presented high fracture toughness and good plasticity and stability. This observation corresponded to the finite element calculations, where α to β morphology was predicted to be coarser for higher holding temperatures performed by Katzarov *et al.*^28^

For two phase titanium alloys, α phase is the basic phase of the (α + β) type titanium alloy, and the number, shape and size of α phase determine the properties of the titanium alloy. In the two phase region, (α + β) phase acquired by heat treatment with holding temperature below the phase transition temperature is characterized by irregular grain shape, continuous and discontinuous α phase and small secondary α phase in the grain boundary, as well as many punctate, spherical, needle, flake and rod like α phase distribution in crystal.^29^

**Fig. 4:**
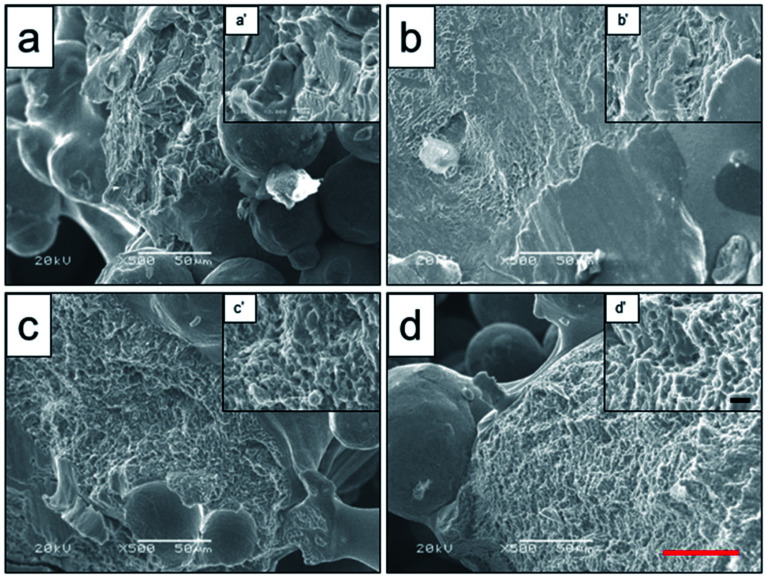
Microstructure analysis of fracture face of original scaffold (a), and scaffolds heat-treated at 800 °C (b), 950 °C (c), 1000 °C (d) obtained by SEM, and the a′–d′ were high-magnification images of the interested zones. Black scale bar = 5 μm and red scale bar = 50 μm.

For analyzing the relationship between plastic fracture characteristics and microstructure of the scaffolds after heat treatment, the microstructure analysis of scaffold fracture face was obtained by SEM (Fig. 4). It showed that the original Ti–6Al–4V titanium alloy scaffolds were composed of lamellar microstructure, and the phase particles were not observed (Fig. 4a and a′). When the scaffold was heat-treated at 800 °C, the fracture appearance was found to be dimple, and the fracture was broken along the lamellar phase (Fig. 4b and b′). When the heat treatment temperature continued to rise (950 and 1000 °C), the crystalline phase increased remarkably, while the grain size increased to sub-micron level with average grain size of 800 nm. Combined with the result of XRD, the crystalline phase was TiAl solution phase in the form of fascicular net structure.

Heat treatment can reduce or eliminate continuous α phase in the grain boundary, and significantly improve the mechanical strength, such as compressive strength, shear strength as well as fatigue strength.^30^ In the process of impact fracture experiment, the hole would form in the original phase and the boundary of the conversation microstructure. With the rise of impact force, these holes got bigger along the boundary of phase before the β phase was across the cluster. The microstructure of mixture phase grew up comparing to the hole. The crack extension had the blocking effect. Therefore the mechanical property was affected by its shape, distribution, dimension and so on.^18^

**Fig. 5:**
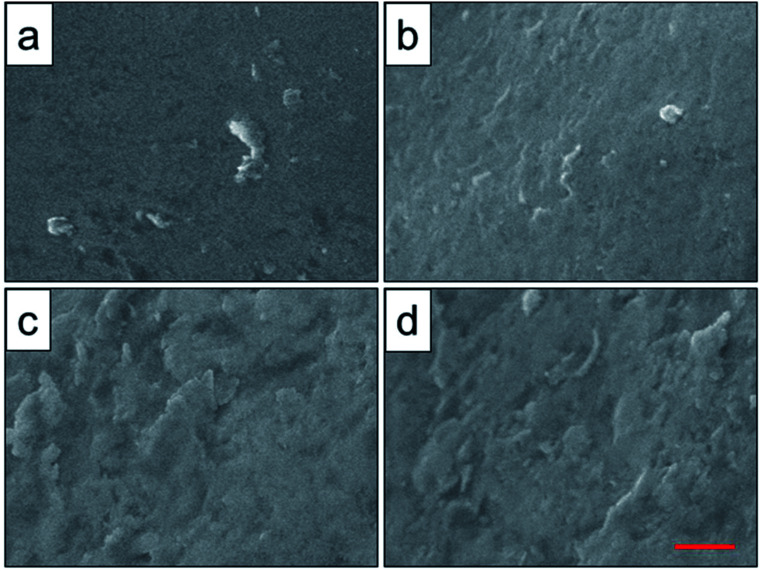
Surface roughness observation of original scaffold (a), and scaffolds heat-treated at 800 °C (b), 950 °C (c), 1000 °C (d) obtained by SEM. Red scale bar = 10 μm.

**Fig. 6:**
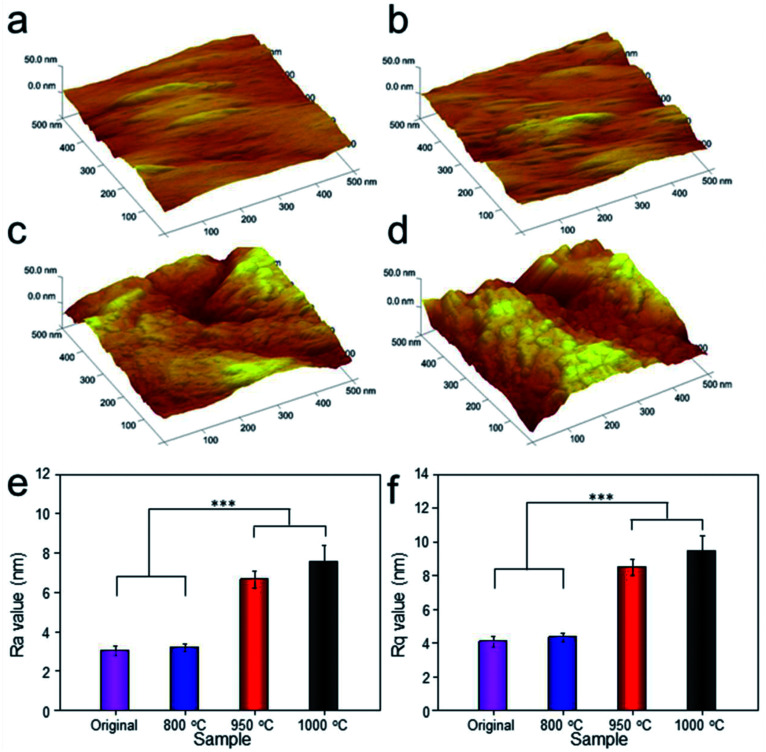
Topological morphology observation of original scaffold (a), and scaffolds heat-treated at 800 °C (b), 950 °C (c), 1000 °C (d) obtained by AFM. *R*_a_ (e) and *R*_q_ (f) values were presented. Data were presented as mean ± standard deviation (*n* = 3, ***P < 0.01).

To analyze the relationship between surface roughness and heat treatment, the change of scaffold surface was observed by SEM (Fig. 5). It could be seen that the original scaffold behaved frictionless surface. When the scaffold was heat-treated at 800 °C, an indistinctive unsmooth surface was observed. Furthermore, more rough and uneven surfaces were detected, when the heat treatment temperature rise to 950 °C and 1000 °C. The topological morphology and quantitative analysis of surface roughness were also obtained by AFM and presented in Fig. 6. It was in good agreement with the SEM images revealing an obvious surface roughness change when processed by heat treatment, especially at 950 °C and 1000 °C (Fig. 6a–d). Furthermore, the *R*_a_ and *R*_q_ values of the scaffolds heat-treated at 950 °C (6.65 ± 0.44 nm and 8.50 ± 0.48 nm) and 1000 °C (7.55 ± 0.83 nm and 9.46 ± 0.92 nm) were significantly higher than those of the original scaffold (3.04 ± 0.25 nm and 4.08 ± 0.33 nm) and scaffold heat-treated at 800 °C (3.20 ± 0.19 nm and 4.33 ± 0.25 nm). This phenomenon could be explained by the oxide layer formation caused decarburization during water quenching, and another possible explanation for roughness change was phase transition and plastic deformation, which also could increase the roughness of the titanium alloy surface.^31,32^

**Fig. 7:**
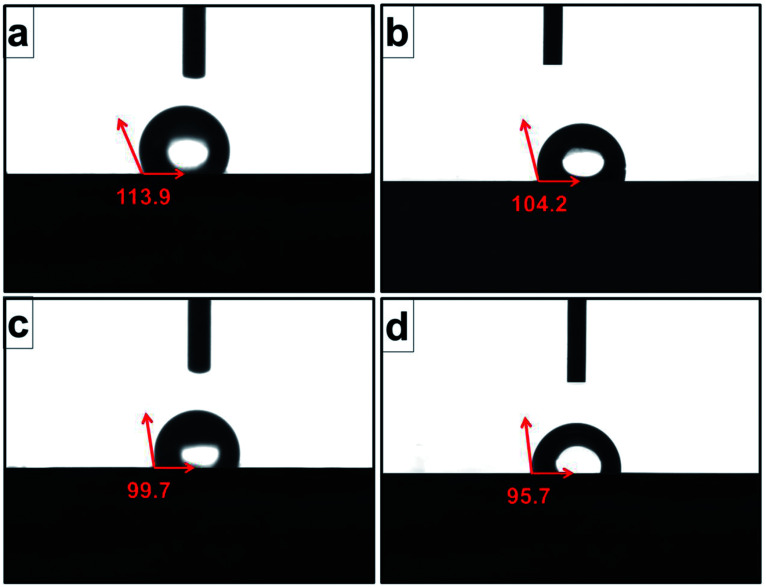
Contact angles of original scaffold (a), and scaffolds heat-treated at 800 °C (b), 950 °C (c), 1000 °C (d) obtained by static sessile-drop technique.

Contact angle value, which was an important index for cell attachment capacity of scaffold, was obtained to evaluate the wettability of scaffold.^24^ As shown in Fig. 7, the average water contact angles for original scaffold, and the scaffolds heat-treated at 800 °C, 950 °C, 1000 °C were 113.9°, 104.2°, 99.7°, and 95.7° respectively. It indicated that increasing treatment temperature could decrease contact angle and improve the wettability of the scaffold surfaces.

### 3.3. Mechanical analysis of scaffolds by heat treatment

Mechanical analysis was investigated, and the result showed that the shear strength and compressive strength could increase gradually when possessed by heat treatment as the temperature rising. In which, the shear strengths of the original scaffold, and scaffolds heat-treated at 800 °C, 950 °C, 1000 °C were 28.64 ± 3.62, 29.65 ± 4.13, 33.98 ± 2.75, and 36.87 ± 3.82 MPa, respectively. While, the compressive strengths of the original scaffold, and scaffolds heat-treated at 800 °C, 950 °C, 1000 °C were 74.74 ± 3.67, 76.87 ± 2.84, 81.39 ± 4.14, and 85.78 ± 3.97 MPa, respectively. This phenomenon could be explained by the transformation of lamellar structure into net structure as demonstrated by optical structure analysis. Meanwhile, transition of α-TiAl to β-TiAl and precipitation of β-TiV could also improve the plasticity and stability of materials by blocking cavity growing and cracks extension.^18^

### 3.4. Response of cells to scaffolds by heat treatment

**Fig. 8:**
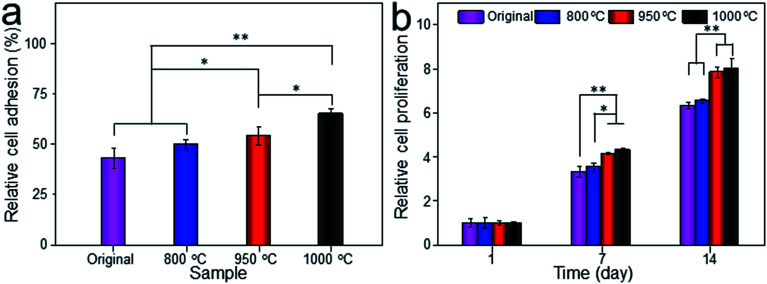
Relative cell adhesion of BMMSCs on the porous titanium cultured *in vitro* for 24 hours with TCP as control (a), and the proliferation of BMMSCs on the porous titanium cultured *in vitro* for 1, 7 and 14 days with TCP as control (b). Data were presented as mean ± standard deviation (*n* = 3, *P < 0.05, **P < 0.01).

Cell adhesion is a complicated process involving several surface features, including the topographic morphology, phase state and surface roughness. The relative adhesion rates of BMMSCs on the original scaffold, and scaffolds heat-treated at 800 °C, 950 °C and 1000 °C were 43.07%, 49.87%, 54.13% and 65.02%, respectively (Fig. 8a). The result indicated that the scaffold heat-treated at 1000 °C (marked as 1000 °C scaffold, similarly hereinafter) showed the best cellular adhesion capacity and the 950 °C scaffold performed better than original and 800 °C scaffolds as a substrate to support the culture of BMMSCs. While, cellular adhesion capacity between original and 800 °C scaffolds showed no significant difference (*P* > 0.05). A previous study showed that the initial cell adhesion force was improved on a Ti–6Al–4V alloy subjected to heat treatment due to surface phase and improved surface roughness.^33,34^ More importantly, this adhesion was considered to be a critical prerequisite for cell proliferation.^35^ As shown in Fig. 8b, the 1000 °C and 950 °C scaffolds exhibited a higher proliferation rate than those of original and 800 °C scaffolds during the incubation period of 7 days, and the gap continued to grow at the incubation period of 14 days.

**Fig. 9:**
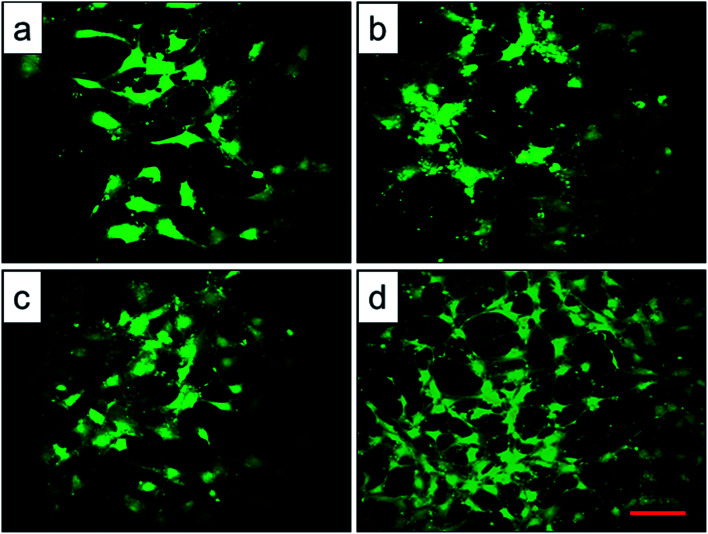
Representative microscopy images of BMMSCs on the top of the original scaffold (a), and scaffolds heat-treated at 800 °C (b), 950 °C (c), 1000 °C (d) observed by fluorescence microscope, scale bar = 50 μm.

To assess the potential of the scaffold structures (configuration of porosity, interconnectivity, surface area) to interact effectively with cells, BMMSCs were seeded and their morphologies were assessed after 24 h culture. Fluorescent imaging indicated discrepant adhesion of BMMSCs to the scaffolds and shared the same tendency with the outcome of cellular adhesion (Fig. 9). It was clearly to be observed that 1000 °C scaffold exhibited the highest cellular adhesion with spindle-like morphology, and the 950 °C scaffold behaved medium cellular adhesion with a mixture of elliptical and spindle-like morphology. While, the BMMSCs on the original and 800 °C scaffolds basically behaved elliptical morphology with low cellular adhesion. Here, we further demonstrated the importance of the surface roughness and surface phase particles for cell adhesion, subsequent cell proliferation as well as spindle-like extension.

### 3.5. Bone ingrowth and osseointegration performances *in vivo*

**Fig. 10:**
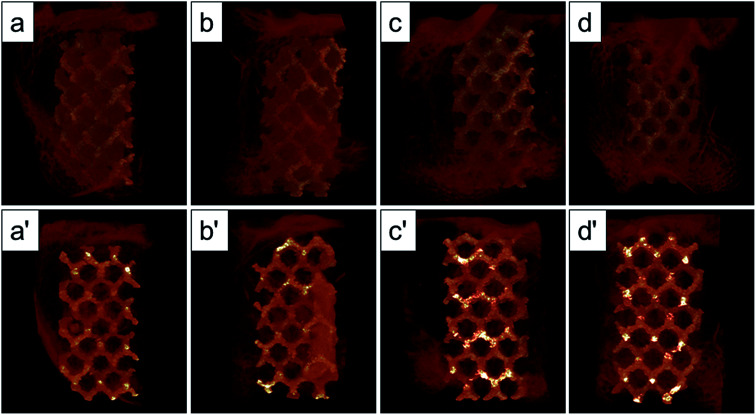
Micro-CT images of the surface layers (a–d) and center layers (a′–d′) of the original scaffold, and scaffolds heat-treated at 800 °C, 950 °C, 1000 °C implanted in the rabbit femur at 12 weeks, and the red color component was bone formed around and into the scaffolds.

**Fig. 11:**
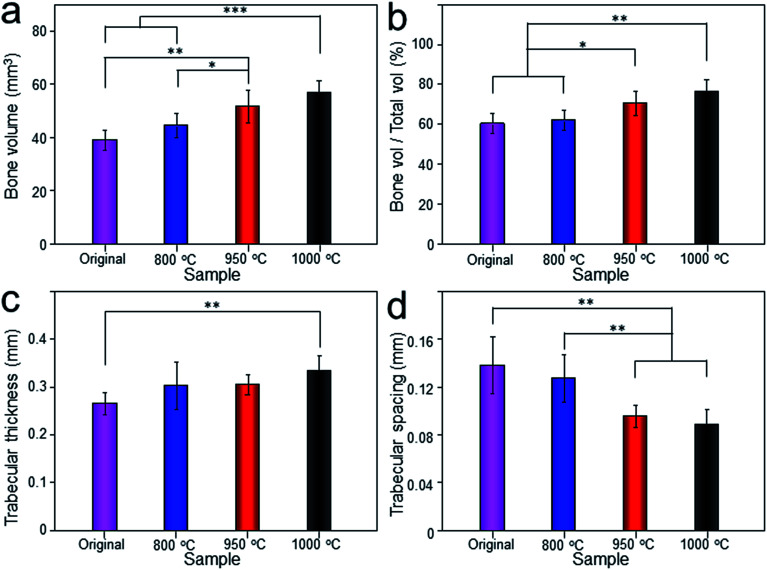
The quantitative analysis of bone volume (a), bone volume/total volume (BV/TV), (b), trabecular thickness (c) and trabecular spacing (d) of original scaffold, and scaffolds heat-treated at 800 °C, 950 °C, 1000 °C evaluated depending on Micro-CT. Data were presented as mean ± standard deviation (*n* = 3, *P < 0.05, **P < 0.01, ***P < 0.001).

The Micro-CT was used to evaluate the new bone formation of the porous titanium implant. It was clear from the 3D reconstruction images of surface layers and center layers that the bone formations of the 1000 °C and 950 °C scaffolds were better than the original and 800 °C scaffolds (Fig. 10). Bone volume and BV/TV ratio of the scaffolds shared the same tendency with 3D reconstruction images. However, no significant difference was detected between the original and 800 °C scaffolds (*P* > 0.05). While, the result of trabecular thickness showed that only 1000 °C scaffold was superior to that of the original scaffold, and the differences between other groups were not statistically significant. Furthermore, the result of trabecular spacing indicated that 1000 °C and 950 °C scaffolds behaved less spacing between the adjacent trabecular bones compared with the original and 800 °C scaffolds (Fig. 11).

**Fig. 12:**
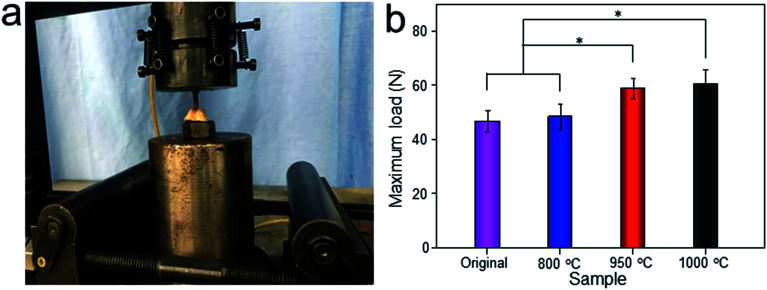
Representative photo of detaching test process (a), and maximum load in the detaching test of original scaffold, and scaffolds heat-treated at 800 °C, 950 °C, 1000 °C after implantation for 12 weeks (b). Data were presented as mean ± standard deviation (*n* = 3, *P < 0.05).

As depicted in Fig. 12, the detachment maximum loads for each scaffold at 12 weeks after implantation were summarized. The 950 °C and 1000 °C scaffolds demonstrated significantly higher fixation strength (58.83 ± 3.78 and 60.37 ± 5.31 N, respectively) indicating stronger osseointegration. By contrast, the original and 800 °C scaffold showed a lower push-out load (46.57 ± 4.76 and 48.26 ± 5.31 N, respectively) indicating weak osseointegration.

**Fig. 13:**
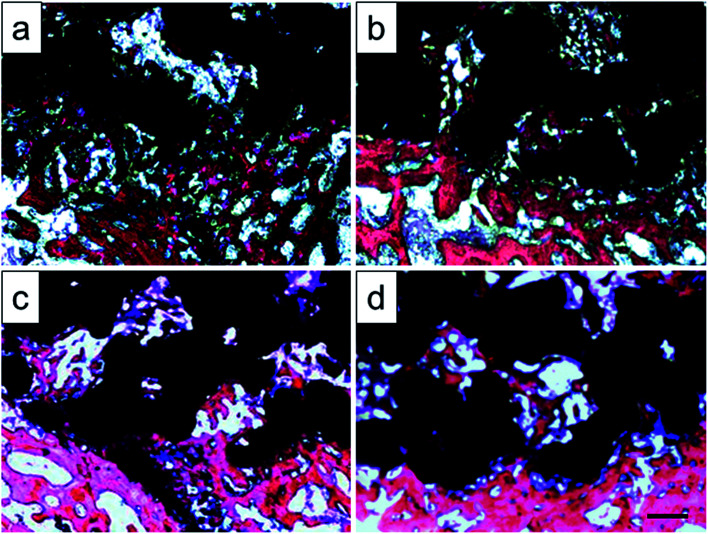
Histologic sections of original scaffold (a), and scaffolds heat-treated at 800 °C (b), 950 °C (c), 1000 °C (d) implanted into rabbit tibia by Van Gieson's picrofuchsin. Purple indicates bone and silver indicates the titanium implant, scale bar = 500 μm.

**Fig. 14:**
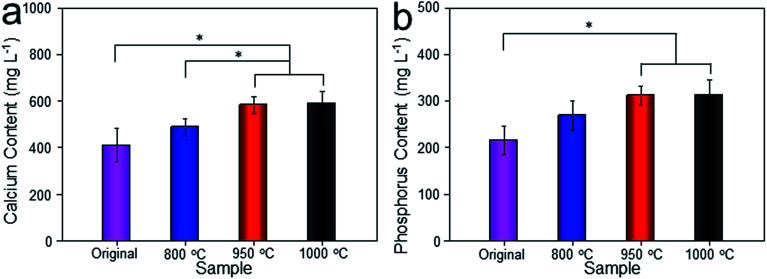
Calcium (a) and phosphorus (b) contents of regenerated bone tissues from original scaffold, and scaffolds heat-treated at 800 °C, 950 °C, 1000 °C. Data were presented as mean ± standard deviation (*n* = 3, *P < 0.05).

Histological images around the bones and implants of each group were shown in Fig. 13. As depicted, newly formed bone tissues and limited inflammatory cells were observed in each sample, indicating good biocompatibility of Ti–6Al–4V porous scaffold. Interestingly, limited osteocytes companied with weak extracellular matrix (ECM) deposition and massive soft tissue were observed in the peripheral pores of the original and 800 °C scaffolds with small part of bone tissue filling the inner pores. However, more new osteocytes concentrated at the peripheral pores of the 950 °C scaffold with no soft tissue filling. The phenomenon was more obvious in 1000 °C scaffold with more inner pore spaces were occupied by bone ingrowth compared with other groups. To quantitatively detect the ECM deposition, Ca and P contents of the regenerated bone tissue were evaluated (Fig. 14). The result showed that Ca and P contents of the 1000 °C (592.08 ± 51.35 mg L^−1^ and 313.26 ± 31.21 mg L^−1^) and 950 °C (583.23 ± 34.12 mg L^−1^ and 312.29 ± 20.26 mg L^−1^) scaffolds were significantly higher than those of in original (410.33 ± 71.97 mg L^−1^ and 215.87 ± 31.63 mg L^−1^) and 800 °C (489.66 ± 36.20 mg L^−1^ and 269.47 ± 30.81 mg L^−1^) scaffolds, which may was attributable to bone or fibrous tissue regeneration differences.

In terms of bone regeneration, 950 °C and 1000 °C scaffolds showed a significantly higher rate of bone formation and higher levels of osseointegration when compared with the original and 800 °C scaffolds, which were demonstrated by 3D reconstruction and mechanical analysis as well as histological examination, synchronously. The results of animal experiments basically correlated with the above-mentioned *in vitro* findings. It could be explained that the improvement of cell adhesion and proliferation enhanced the attachment of endogenous osteoblasts and BMMSCs, which might lead to better bone ingrowth and osseointegration.^36^ Another possible explanation for the better mechanical stability of 950 °C and 1000 °C scaffolds as compared to the original and 800 °C scaffolds, which could be the better interface connection between the regenerated bone tissue and scaffold. These features might result in better (mechanical) interlocking of the regenerated tissue with the implanted materials and thereby strengthen the interface between the regenerated bone and the titanium.^37^

Porous titanium alloys have great advantages in orthopedic implants because of the biocompatibility and porous structure, which provide an ideal environment for bone ingrowth.^38^ Previous studies have proved that 3D printed porous Ti–6Al–4V implants could partly promote bone ingrowth and osseointegration.^39,40^ However, filling the bone defects with porous titanium alloys alone shows limited effect on bone ingrowth and osseointegration.^41^ Increasing the porosity of Ti–6Al–4V scaffolds can promote the bone ingrowth, however, it also greatly decreases mechanical strength of scaffolds.^42^ Hence, obtaining an appropriate bone ingrowth/strength balance of the biomaterial is of great significance. Herein, we investigated the influence of heat treatment on the properties of Ti–6Al–4V porous titanium alloy. Heat treatment was able to improve mechanical properties of scaffolds significantly, and then enhanced bone ingrowth. This is important for porous titanium alloys to maximum advantages in orthopedic metal implants.

## 4. Conclusions

With the increase of solution treatment temperature, Ti–6Al–4V prepared by EBM underwent phase changes from TiAl to TiAl + TiV. During solution treatment at 1000 °C, α phase with lamellar arrangement distributed in the metastable β phase, and then formed a net organization in β phase. The net organization presented high fracture toughness and good plasticity and stability. These properties met the requirement of medical implant and also were demonstrated by mechanical strength test. Besides, the process of heat treatment increased the surface roughness of porous titanium alloy, in addition of influence of surface phase, which promoted the early BMMSCs adhesion rate and thus affected the later proliferation behavior. Furthermore, the animal experiment also demonstrated that the scaffolds after heat treatment exhibited better bone ingrowth than the original ones.

## Conflicts of interest

There are no conflicts to declare.

## Supplementary Material
